# Material Environments and the Shaping of Anorexic Embodiment: Towards A Materialist Account of Eating Disorders

**DOI:** 10.1007/s11013-021-09715-8

**Published:** 2021-04-07

**Authors:** Karin Eli, Anna Lavis

**Affiliations:** 1 Division of Health Sciences, Warwick Medical School, University of Warwick, Coventry, UK; 2 Unit for Biocultural Variation and Obesity, School of Anthropology and Museum Ethnography, University of Oxford, Oxford, UK; 3 Institute of Applied Health Research, Institute for Mental Health, University of Birmingham, Birmingham, B15 2TT UK

**Keywords:** Anorexia nervosa, Eating disorders, Materiality, Embodiment, Ethnography, Mental health

## Abstract

Anorexia nervosa is a paradoxical disorder, regarded across disciplines as a body project and yet also an illness of disembodied subjectivity. This overlooks the role that material environments—including objects and spaces—play in producing embodied experiences of anorexia both within and outside treatment. To address this gap, this paper draws together two ethnographic studies of anorexia to explore the shared themes unearthed by research participants’ engagements with objects that move across boundaries between treatment spaces and everyday lives. Demonstrating how the anorexic body is at once both phenomenologically lived and socio-medically constituted, we argue that an attention to materiality is crucial to understanding lived experiences. A materialist account of anorexia extends the literature on treatment resistance in eating disorders and offers a reconceptualisation of ‘the body in treatment’, showing how  objects and spaces shape, maintain, and even ‘trigger’ anorexia. Therefore, against the background of the high rates of relapse in eating disorders, this analysis calls for consideration of how interventions can better take account of eating disordered embodiment as shaped by material environments.

## Introduction

Anorexia nervosa is a seemingly paradoxical disorder, framed in both sociological and clinical discussions as a body project and yet also as an ethereal illness of subjectivity. Across the many disciplines that engage with anorexia—psychiatry, psychology, sociology, anthropology, history, philosophy and neuroscience, among others—reflections on meaning, subjectivity and embodiment have tended to hinge upon the emaciated body. This has been held up as the focal ‘object’ of analysis as well as assumed to be the locus of desires and practices in anorexia.

Yet, such a reification of emaciation as a ‘synecdoche’ of anorexia (see Lavis 2011), serves to position the body as an empty vessel ready to absorb and display cultural discourses. This situates individuals with anorexia as susceptible to cultural influences while also depoliticizing and individualizing their experiences (Saukko 2000; Malson and Ussher 1996), framing these as the products of pathologized selves (Lester 1997). Moreover, as Bray (1996) has argued, the construction of the anorexic body as a non-agentic vessel for competing (popular and scholarly) cultural discourses is inextricably linked to the feminization of this disorder, with women cast as vulnerable, uncritical recipients of culture writ large (see also Burke 2006). In social theory, for example, the feminine anorexic body has been situated as a cultural artefact—a blank slate, inscribed with the repressive discourses of patriarchal religion, power structures, and Western philosophy (Bray 1996; see, e.g., Bordo 1993; Heywood 1996). This analytic strain has led to a pervasive cross-disciplinary practice of reading meaning retrospectively from the visuality of the starved body, thereby ‘colonizing’ the gazed-at body with external meanings (Bray 1996), and dualistically privileging an individual’s body over their voice or embodied practices (see Lavis 2018). Such a reading arguably ignores the material dynamics of lived experience and embodied practices.

It has long been known that anorexia is connected with identity (Bruch 1973; Bardone‐Cone, Thompson, and Miller 2020). It is often egosyntonic and may even be valued by the person living through it (Schmidt and Treasure 2006; Lavis 2018). In exploring the “everyday worlds of anorexia” (Warin 2010) and ways of “being anorexic” (Gooldin 2008) recent anthropological and sociological analyses have offered ways to (re-)conceptualise such relationships between illness and identity by reflecting on embodied practices. They have explored the “embodied sentience” (Warin 2003, p. 78), “liminality” (Eli 2014a, 2018) and “self-care” through traumatic life experiences (Lavis 2015) that may be embedded in the mundane practices of individuals with anorexia. In so doing, these analyses have cast new light on previous literature that has discussed the ambivalence that individuals with anorexia may feel towards recovery (Tan et al. 2006). In recognising this ambivalence as key to anorexic being-in-the-world (Warin 2010), anthropological and sociological analyses have thereby also illuminated lived experiences of treatment (Lester 2007; Gremillion 2003), which can be both wanted and yet also felt to enforce the distressing loss of an, albeit painful, “friend” (Lavis 2013).

Whilst a fixed definition of recovery is contested by those with lived experiences of eating disorders and scholars (Eli 2016; LaMarre and Rice 2021), according to clinical definitions, fewer than half of patients with either anorexia nervosa or bulimia nervosa fully recover (Schmidt et al. 2016). Individuals with eating disorders have significantly elevated mortality rates. Anorexia, for example, has amongst the highest mortality rates of any mental health disorder (Arcelus et al. 2011) and eating disorders can result in major long-term burdens in morbidity and mortality (Demmler et al. 2020). People with eating disorders often experience relapse after treatment (Khalsa et al. 2017), with those with anorexia, specifically, known to be at extremely high risk of early relapse (Carter et al. 2012). Against the background of recent anthropological and sociological research noted above, to underpin a much-needed improvement in treatment outcomes there remains the need for a new perspective that engages with the thorny intersections of embodied practices, illness and treatment. In suggesting that a materialist perspective can do this, this paper aligns with emerging scholarship that has situated conditions such as obsessive-compulsive disorder and dementia vis-à-vis everyday objects also to forge new understandings of experiences through material engagements (Parish 2018; Malafouris 2019). In the paper we explore the key role that material objects and the spaces they move through play in shaping, framing and disrupting what we term ‘anorexic embodiment’. By ‘anorexic embodiment’ we mean the lived experience of being-in-the-world with anorexia, encompassing everyday practices alongside sensations, perceptions, cognitions, and concepts of identity and self.

### Anorexic Embodiment: A Materialist Approach to Lived Experiences of Anorexia

Whilst discussions of embodied experiences of anorexia in anthropology and sociology have laid the groundwork for the materialist account offered in this paper, across the social sciences, phenomenological analyses of illness have, more widely, been critiqued for foregrounding the body at the expense of materiality and spatiality (Olsen 2003) and this is pertinent to reflections on anorexia. Although the embodiment paradigm (Csordas 1993), like other phenomenological approaches (Desjarlais and Throop 2011), posits that human bodies are entangled in co-constitutive, inter-subjective connections with other human and non-human subjects, objects, and spaces, in practice, phenomenological analyses have largely relegated material environments to the backdrop of human experience and interaction (Olsen 2003). This has also been the case in explorations of anorexic embodiment. While some previous studies have attended to anorexic participants’ sensory experiences of the taste and texture of foods (Warin 2003; Lavis 2013, 2016) and of material objects associated with cleaning, self-measurement, and self-harm (Warin 2010), none has positioned material objects at the centre of analysis. To do this calls for an approach that draws on a wider set of analytic tools than previously utilized in discussions of eating disorders. In particular, it necessitates an engagement with the recent materialist turn across the social sciences and humanities (Miller 2010).

Over the past three decades, the materialist turn has focused on the ‘stuff’ (Miller 2010) of human and non-human environments and bodies. Although united by a core interest in material culture, the various disciplinary strands of this engagement have taken differing theoretical approaches to objects: from exploring social life through a material lens (Miller 2010; Miller and Woodward 2012), to advocating a “radical essentialism” which breaks down divisions between concept and matter, object and subject (Henare, Holbraad, and Wastell 2007). All have, however, challenged constructivist theorisations of sociality that leave intact a previously assumed distinction between material and social processes. A materialist lens has thereby emphasised the need to recognise the “active processes of materialization of which embodied humans are an integral part, rather than the monotonous repetitions of dead matter from which human subjects are apart” (Coole and Frost 2010, p. 8). For example, the concept of “embodied space,” as articulated by Setha Low (2003), militates against the delimiting of body from geography and of social structure from physical structure.

Of particular relevance to this paper are recent explorations of materialities of care in the sociology of health and illness. These have highlighted that an attention to mundane objects and spaces is central to understanding embodied experiences (Buse, Martin and Nettleton 2018). As Brownlie and Spandler (2018) have suggested, attending to the materialities of the everyday can bring into view the small-scale dynamics that populate lived experiences of providing, accepting, and denying care. Moreover, the “quiet” materialities of healthcare, such as gloves or soap, may be taken for granted in the fabric of the clinical everyday but are crucial to shaping the experiences of those receiving care (Pink, Morgan and Dainty 2014). Interrogating relationships between such materialities and care practices has thereby offered insights into how the materialities of treatment practices forge illness and recovery experiences.

### Bodies, Objects, and Spaces: Applying a Materialist Lens to Ethnographic Data

Against this theoretical background, we argue that material environments—meaning objects and spaces—are crucial to anorexic embodiment. Bringing together separate ethnographic fieldwork projects, which were carried out in Israel (Eli:2005–2006, 2011) and the UK (Lavis:2007–2008), the authors explore the materialities of anorexia by focusing on five objects that were, each in a different way, described by participants as core to their everyday experiences of anorexia: a photo album; a chair; a television set; biscuits; and paperback memoirs of anorexia. The authors decided to examine their datasets together due to this resonance of material objects in their respective field sites. Through discussing the role of materiality in their fieldwork observations, the authors recognised that, while different material objects emerged as prominent in each field site, reflecting local structures, practices, and concerns, when brought together, these objects spoke of broader experiences of anorexia as forged at the intersections of treatment and everyday life. As such, the authors recognised that analysing their data jointly could shed new light on anorexic embodiment.

Positioning this analysis of anorexic embodiment both in and out of treatment is argued to be crucial to understanding lived experiences. In Israel and the UK, where the authors conducted their fieldwork, living with a diagnosis of anorexia almost always involves living with outpatient or inpatient treatment, however desired or resisted. This is due both to the local nationalised healthcare systems, and to the position of anorexia within these systems, i.e., as the eating disorder considered most pressing, due to the significant medical complications of starvation (Gibson, Workman, and Mehler 2019) and the frequent distress of informal carers (Treasure and Nazar 2016). We acknowledge that this trajectory from diagnosis to treatment is country-specific, and is not shared in managed care contexts, such as in the US healthcare system (see Lester 2019). Within the fieldwork contexts discussed in this paper, however, lived experiences of anorexia are arguably intertwined with treatment categories, practices, and materialities. What we term in this paper anorexic embodiment is, therefore, forged at the intersections of the clinical and mundane.

Key to the present paper, thus, is that materialities can trouble or “open up breaches in the categories we use to order the world” (DeSilvey 2006, p. 321)—in this case, categories, both clinical and cultural, used to define anorexia and the people who live through it. Further to this, we also argue that objects fundamentally produce lived experience both in and out of treatment; these objects, however hidden or taken-for-granted, participate in the everyday shaping of anorexic bodies and worlds. By approaching them as “mutable artefacts” (DeSilvey 2006, p. 325) that forge body and being, the paper draws into view the “material dynamics of embodiment” (Lavis and Eli 2016) in anorexia.

## Methods

### Eli

The ethnographic case studies that centre on an Israeli eating disorders inpatient ward (2005–2006) were part of Eli’s doctoral research. The study, which has been described in detail elsewhere (Eli 2014b), focused on the illness and recovery narratives of 36 Jewish Israeli participants (35 women and one man) with past or present eating disorders (anorexia nervosa, bulimia nervosa, or eating disorder not otherwise specified). The study was mainly community based, and about a third of the participants had been hospitalized previously in the inpatient ward. Participants were recruited through three main sources: an informal network led by the study’s key participant; an eating disorders clinic, where staff members recruited past and present patients^1^ whom they deemed well enough to participate; and a Hebrew-language eating disorders online discussion board. The study received ethical approval from the University of Oxford’s Central University Research Ethics Committee and from an Israeli healthcare fund Helsinki ethics board. Study participants provided signed informed consent.

### Lavis

The ethnography from the English eating disorders inpatient unit (2007–2008) derives from Lavis’ doctoral research. 45 patients took part in the ethnography. Forty staff members also participated in the study; however, this paper does not refer to the data collected through interviews with or observations of staff members. From both groups, informed written consent to ‘hang around’ as an ethnographer was gained, as well as additional informed written consent for each interview. The focus of this research was to explore everyday experiences of anorexia and, specifically, to interrogate the desire amongst some individuals with anorexia to maintain their existing illness. Thematic analysis (Braun and Clarke 2013) was conducted on interview transcripts and ethnographic fieldnotes. The research received ethical approval from the National Health Service.

All names used in this paper across both studies are pseudonyms.

While these ethnographic studies were conducted in the mid-to-late 2000s, both authors’ recent and ongoing research in the area of eating disorders (see Eli 2018; Lavis 2018) has confirmed that the ethnographic material presented in this paper continues to be relevant. Eating disorder inpatient treatment regimes in each country have largely remained consistent, such that the material engagements described in this manuscript are as pertinent now as they were when the authors’ earlier ethnographic studies took place.

## Resistance and Relationality: Ward Space to Body Space

First, we present two case studies drawn from Eli’s fieldwork among people with eating disorders in Israel. While this fieldwork was community based, rather than clinically based, medical institutions featured centrally in the participants’ narratives. Either prior to or during the time of the study, all 36 participants had some form of clinical care, including treatment in outpatient eating disorders clinics, general psychiatric wards, and individual sessions with dieticians, psychiatrists, clinical social workers, and psychotherapists. Fourteen of the participants had been hospitalized in the same eating disorders inpatient ward.

The following two case studies focus on experiences of life on the ward, both remembered and in the present-tense. Episodes from ward life, as Eli learned through the narratives of the fourteen participants who had been hospitalized, continued to resonate beyond hospital walls, accreting into autobiographical reference points. Unconfined to a bounded time and space, ward materialities carried into the participants’ recovering/recovered being: the blue measuring cups, used in the ward dining hall to apportion carbohydrates and vegetables, transitioned, in replica, into a participant’s kitchen and daily cooking practice; the ward’s calorific concoction—cornflakes with dairy custard—reappeared in another participant’s university life, when she recognized a fellow student as a former inpatient based solely on her consumption of this dish at the university café. These materialities, heightened through their emplacement in an authoritative clinical space, continued to articulate anorexic embodiment outside the ward. Participants’ narrations of and interactions with ward materialities delineated an anorexic embodiment forged through contradictions and ambivalences, as material objects became extensions of the (formerly diagnosed and treated) self, growing to ‘contain multitudes’ (Whitman 1975[1855], p. 123) of compliance and resistance, illness and health, belonging and difference (Eli 2014a).

### Grace and the Photo Album: Remembrances of Ambivalence

Grace had a photo album dedicated to her life on the ward. She began to take photographs three months after she was hospitalized, keeping a detailed record of the people, spaces, and objects that marked her stay. A typical stay in inpatient care entailed weeks or months of living in the ward. Patients who showed progress were allowed to go home for weekends or to spend a weekday afternoon away; for the most part, however, inpatient stays involved continuous living in the ward’s enclosed space, with the occasional supervised excursion to the ward’s front yard, or a guided walk at night to a nearby landmark. Life on the ward was marked by the strict schedules and regimens of diet and activity prescribed by the senior clinicians, under the observation of nursing staff.

Early on during Eli’s fieldwork, and following several interview hours, Grace offered to show her the photo album—the object that materialized Grace’s stories about the eating disorders ward, providing a visible and tangible illustration of what she had described. This object served as Eli’s initiation into the ward, which, at that point, she had not yet visited. Guiding Eli through the album, Grace filled in the relational gaps in her photographs: identifying roommates, best friends, and favourite members of staff, telling Eli who had since given birth, and who was no longer in touch. Grace photographed and narrated the ward with a loving inflection, and as she turned the pages—in her kitchen, years after she had been discharged—she did so with a tinge of nostalgia. “It’s fun, nice, a sweet memory”, she reflected; “but I don’t miss it anymore… I used to miss it so much”.

Grace’s photographs and stories, while positive, continued to highlight experiential moments that designated the ward as a space of surveillance and resistance: there was the common room (or as the ward management named it, ‘the day room’), where patients were watched for two hours after every meal; there was the bathroom, where patients were followed by nurses, who stood watch; and there was Grace’s room, with her suitcase perpetually packed. “I was always with one foot out”, she explained. This image, however, did not conflict with her story of saying lingering goodbyes, on her last day at the ward, having been discharged but feeling unable to leave.

The ward, as it emerged in Grace’s account, was a haven. “It was,” she said,

a little lab like that, that you could be inside…. A lab in the sense that it was very sterile, it was – very very exact and measured conditions, and – you knew that you, it’s not like the real world, so it eased [our burden].^2^

But the ward, as Grace later explained, was also a prison. As she went through the album, and Eli observed that Grace had parenthetically noted the diagnosis of each fellow patient she had photographed, she explained that “it was so I’d remember”. Eli asked her why, and she said, her voice somewhat tense, “I defined the disease [each patient] was in, and she also didn’t have a problem defining the disease she got, it’s not—I didn’t define it judgmentally or divisively, I wrote it in the sense of noting a fact. She got in—*what is she in for*? For bulimia. Okay, great”. Grace and Eli then began laughing, and Grace said that clarifying what each patient was ‘in for’ was part of every new arrival. The ward may have been a haven—a space away from “the real world”—but Grace kept records that testified to the authoritatively structured, carefully surveyed grids of sociality that the ward entailed. Her photo album, then, evoked ambivalence across time: both the ambivalence she had experienced as an inpatient, years ago, and the ambivalence she experienced in the present tense, when recollecting, narrating, and making sense of her past patienthood. Leafing and talking through the album’s pages, Grace opened up the co-existing tension and wistfulness she associated with her inpatient experiences, communicating her still-ambivalent relationship with the anorexic identity forged on the ward.

### Sara, the Chair, and the Television Set: Multiplicities of Resistance

Eli visited the ward for the first time in the summer of 2006, several months after being introduced to the eating disorders ward, and the ambivalence therein, through Grace’s photo album. Sara, a participant with whom Eli had been in contact since her fieldwork began, entered the ward as an inpatient, and Eli visited her regularly. Through these visits, she witnessed Sara’s everyday enactments of compliance and resistance. When Eli first visited, she met Sara in the common room, where most patients congregated (with or without visitors), and where the main features were a flat-screen television, a few desktop computers, and many upholstered chairs. With the guard at the door—a clinical social work or nursing student—keeping watch, patients (and, by association, visitors) were engaged in the task of sitting still. It was the two-hour surveillance window after the patients’ mid-afternoon snack, and Sara, like other patients classified as anorexic, was not allowed to move around. Although the chair might have seemed incidental to Sara’s actions, it was, in fact, a crucial actor in the scene that unfolded. In the common room, the chair was transformed from a piece of furniture into a clinical object, used to confine Sara’s rebelling body after each meal. In conversation with Eli and her aunt, observed by the watchful guard, Sara made sure to stay seated. Occasionally, however, she pretended to yawn, stretching her arms at length while keeping her torso firmly within the confines of the chair. Each furtive stretch ended with an exchange of knowing smiles; she and Eli were acknowledging the resistance embodied in each subtle, and forbidden, stretch. But Sara complied with the rules, otherwise—eating every meal, drinking water only when allowed, and honouring her clinicians’ trust when they sent her for an afternoon away with the instruction to eat an ice cream bar (which she did).

Sara’s resistance also took on less obvious shapes and targets. During a later visit, when Sara and Eli were engaged in conversation, another patient announced excitedly that her sister had just texted her to say that ‘Hunger Point’ (Silver, 2003) was being broadcast. The patient turned the television on, and images of pro-anorexia website texts and photographs immediately flooded the screen. This was no surprise—‘Hunger Point’ is a made-for-television film in which a young woman dies of anorexia (immediately after being released from a residential treatment centre), and her sister then turns to pro-anorexia websites for comfort. Sara was incensed. She angrily got up from her chair and turned the television off. ‘But why?’ the other patient protested; ‘this is a film about a girl who overcomes the disease!’ Watching ‘Hunger Point’ in the common room was permitted, under ward rules—as was watching the Israeli version of ‘The Biggest Loser’, in which contestants classified as obese engage in gruelling physical activity and weight-loss competitions (watching this show, according to Sara, was some patients’ favourite pastime during post-dinner surveillance). But Sara told Eli she couldn’t take it—being surrounded by eating disordered media and by patients’ incessant anorexic talk.

Sara’s resistance was directed at more than avoiding the triggering effects of anorexic talk and pro-anorexic images. On the ward, other patients were never fully ‘other’; ward life blurred the boundaries of selfhood, creating images of oneself—past, present, and future—that had equal power to inspire and to horrify (Eli 2014a). As such, Sara’s possibilities for the translation of others’ bodies, words, and practices were limited due to the confined spatial and relational configurations afforded by the ward. Thus, the scenes she witnessed shape-shifted into a deterministic, future image of herself. Such instances of identification heightened the danger that inhered in other patients’ enactments of disorder. Although Sara did not resist the idea that she would be anorexic for the rest of her life, she resisted the threat of becoming *all anorexic*. Through mundane objects in the ward’s common room, Sara expressed a dual resistance: small subversions of the treatment regime, embodied in shifting and stretching on a chair meant to confine her movements after meals, alongside protests against fellow patients’ performances of eating disorders, embodied in switching off the television set where they played eating disordered media. With this dual resistance, Sara was continuously engaged in maintaining a fine balance—reasserting her anorexic self, while not losing herself to the patient group, and by extension, to anorexia.

Rather than highlighting the so-called pathology of the anorexic body or self, the engagements with materialities discussed so far have emplaced anorexic embodiment both within and beyond the logics of everyday clinical contexts, at intersections where illness and identity are continuously negotiated, parsing out relationships between self, others, objects, and spaces. Running through the participants’ narratives and practices are the constitutive elements of biomedical power in generating and reinforcing anorexic modes of being-in-the-world, whilst also showing the affordances of objects and spaces to disrupt these. Anorexia, thus, has emerged here as not simply the manifestation of a pathologized relationship between a person and her body but as shaped by material environments.

Anorexic embodiment, then, is seen to be constantly created and recreated through engagements with multiple subjects and objects. The two vignettes so far have shown that it is through the relationships among the self, clinicians, other patients, as well as the spaces and material culture of the ward, that anorexic embodiment is lived, remembered, and defined. Exploring materialities has thereby begun to offer insights into anorexic embodiment as a continual process of drawing and redrawing relationships between illness, corporeality and subjectivity; it is these intersections that will now be drawn to the fore through Lavis’ analyses.

## Selves, Others and Mealtimes: Body Space to Anorexic Space

Taking up the interrelated strands of ambivalence and resistance introduced by Eli’s explorations of the material dynamics of lived experiences of eating disorders treatment, and the porous boundaries of selfhood that these illuminate, Lavis now interrogates two objects that were framed by participants as core to their experiences of treatment and  to anorexia more widely: anorexia memoirs and food—in this case, biscuits. Woven through life within the Eating Disorders Unit (EDU) and beyond it, these emerge as at once utterly mundane and yet clinical, with their material exchange between spaces transacting and disrupting multiple meanings at once. Just as the objects that Eli discussed accrete into autobiographical reference points, biscuits and memoirs draw clinical spaces and categories into the forging of anorexic embodiment in ways that speak of the continual effort it involves both with and beyond treatment.

### Exchanging Memoirs: Disruptive Materialities

In the English Eating Disorders Unit (EDU), it was during quiet moments in the patients’ lounge, as well as the fraught mealtimes of the dining room, that participants’ complex relationships with, and embodiment of, anorexia were made, articulated, and rendered visible. In relation to the very structures of the EDU’s spaces and times, various aspects of these relationships came to the fore or receded, and one moment at which this was most apparent was during the enforced ‘rest periods’ after mealtimes.

Echoing Eli’s discussion of the Israeli ward, in the UK EDU each meal was followed by a rest period of between fifteen and forty-five minutes during which activity was limited and the, usually calm, patients’ lounge became a space of tension and surveillance. By foregrounding the optimization of nutrient absorption as paramount to patients’ temporal, spatial, and bodily routines, rest periods served to designate food as *the* treatment for anorexia, thereby framing anorexia primarily as both *in* and *about* the body (see Lavis 2015). As such, rest periods were described by many participants as “awful.” In the imposed emptiness of dead time, many felt unable to do anything but contemplate what they had just eaten; the guilt and shame of their having “threatened” their anorexia, nourished themselves, or often both, weighing heavily. During Lavis’ fieldwork many participants tried to sleep through rest periods, whilst others read, watched TV, or knitted. If Lavis ever passed through the patients’ lounge during mealtimes it had a blank expectant air, filled with objects that vulnerably exposed the raw intimacies of patients’ lives in the EDU; in neat piles on sofas and floors had been placed pens, paper, notebooks, novels, knitting needles or crossword books by people who knew that their bedrooms would be locked and inaccessible following the meal. Objects that could always be found nestling among sofa cushions, ready to be digested in rest periods, were memoirs of anorexia. And while the advent of smartphones and social media in the last decade has changed modes of communication about eating disorders and mental health more broadly, memoirs have retained their power.

Anorexia memoirs were cited by one participant, Elle, as “inspirational”. She said, “I like to see if they [the authors] do it [anorexia] better than me. It’s inspiring; I want to starve more to be as good as them.” As Eli has highlighted, ward spaces are milieus in which individuals are made and unmade as anorexic in relation to other people—to their bodies, their gaze and their eating practices, for example, as group identity may be entrapping. Through the reading of memoirs these grids of sociality are extended into the textual. Both in and out of the clinic, the narrative of an other’s illness affords a way of ‘remaking’ one’s own anorexia, and this took on particularly urgent resonance for participants at mealtimes and during rest periods. In her interview Kate said, “I read as much about anorexia as I can.” She sometimes read academic texts about the illness, but her preference was for memoirs. Reading memoirs during rest periods, with her feet tucked under the frayed cushions of the sagging sofa as Eva knitted next to her was, to Kate, about re-entering the space—or “bubble”—of her anorexia, which she described as “a friend”. At the very moment at which her anorexia felt most exposed and under threat after the enforced ingestion of food, immersing herself in the anorexia of others through text allowed Kate to strengthen and reclaim her own illness, thereby discursively resisting the food she had just eaten and its clinical meaning as an object of care. In so doing, Kate mobilised her anorexia, placing it beyond her body and, as such, beyond the threatening reach of treatment. Re-entering anorexia through the words of a distant textually-rendered other thereby permitted Kate to connect the points of her life with anorexia in ways that speak of the inherent complexity of anorexic embodiment.

As such, in dialogue with Eli’s analysis, here a materialist lens has begun to challenge a habitual framing of anorexia as centrally *about* an ever-decreasing body. Memoirs-as-objects draw into question such an—often taken-for-granted—relationship between anorexia and the body. Kate’s actions of linking and relinking her body and anorexia through her engagement with memoirs illuminates relationships, but also disjunctures, among anorexia, embodiment, and bodies. These pose challenges to contemporary sociological *and* clinical ways of thinking about this illness and its relationship to corporeality. In drawing the complexities of anorexic embodiment to the fore, engagements with memoirs have demonstrated that while anorexia may be enacted *with* the body, it is not centrally *about* the body. We might even say that, although profoundly and painfully embodied, anorexia is not necessarily bodily, as Lavis has previously argued (see Lavis 2011, 2013). Further, as Lester has suggested, rather than representing ‘facts’ of the body, eating disorders represent dynamic ‘flows’ that speak to cultural concerns with boundaries and relatedness’ (Lester 2019:35).

Crucially, memoirs challenge, rather than channel, simplistic associations between anorexia, resistance, and the consumption of ‘triggering’ images or words. Like the photo album in Eli’s analysis, through which Grace had assembled a project of remembrance, memoirs delineate daily life on the ward, documenting people, objects, and spaces to enable narrative integration following the ‘biographical disruption’ of inpatient treatment (Bury 1982). As such, memoirs set in motion engagements beyond the page. Crucially, in the EDU these books were not just discursive ‘learning tools’ to resist clinical treatment; they were also material objects with particular affordances that came into view through their handling and exchange. The importance of memoirs to participants lay not only in their textual meanings, but also in how they moved as objects through the EDU in ways that disrupted and transformed this space and the bodies and selves within. To be seen to be reading, holding or swapping a memoir during a rest period was an act of resistance, but it was a hidden one. Staff members often spoke about how engaging with others’ stories in this way showed signs of desiring recovery, and they therefore viewed memoirs as materialities of care. Indeed, in the EDU memoirs were sometimes lent to patients by staff to inspire recovery, which implicated these objects into a regime of care, aligning them with the central object-as-care on the ward, which was food. In contrast, like other objects such as calorie counting books, memoirs were often exchanged amongst patients. They too reflected in interviews on these exchanges as “caring” acts, with care defined through their aiding of other patients to hold onto anorexia in the face of the threat and struggle posed by treatment. This movement of memoirs thereby folded new and resistant meanings into the space of the clinic in ways that were quietly ambivalent. For participants, the materiality of the memoir, as an object seen but not seen by staff, became central to a reimagining of space both in and around the body. In line with the other objects focused on in this paper, thus, the meanings ascribed to and enacted by these books remained hidden or were revealed depending on the person interacting with them.

Memoirs, then, have begun to highlight how anorexic embodiment may be continually negotiated and remade through materialities that make meanings as they move through spaces and across boundaries. In so doing, this analysis has joined that of Eli in showing how material objects become mutable as they traverse the blurred boundaries of the clinical and mundane, and how this movement can occur within the seeming singularity of clinical space. This sense of mutability and shifting meanings will be further drawn to the fore in the paper’s final vignette.

### From the EDU to Starbucks: Biscuits as a Space Apart

As the paper so far has shown, materialities may forge anorexic embodiment in the very space designed to unmake this. One object that needs attending to in this context is, therefore, food. This is habitually noted for its absence in anorexia, with self-starvation a core diagnostic tenet of the diagnosis (APA 2013). Yet, exploring the mutable meanings of food as it moves across spaces and through bodies is central to furthering an understanding of practices and lived experiences of anorexia (Lavis 2013, 2016). Although memoirs were seen, above, to disrupt the enforced feeding of treatment by offering Elle, Kate and others a space through which to remake anorexia against the threat posed by food to one’s “friend”, fear and avoidance of food is not the end of the story. Lavis has written elsewhere (2013) that “to (continue to) be anorexic, one must both eat and yet not eat”; this tension is fundamental to anorexic embodiment and is navigated both in the clinic and outside it. As such, any discussion of how a materialist perspective furthers understandings of lived embodied experiences of anorexia needs also to consider food as an object. This assertion intersects with recent work on the material entanglements of food and bodies, which has explored relationality among human and non-human others through the prism of food (Abbots and Lavis 2013; Ellis 2018). Specifically, Lavis, Abbots, and Attala (2015) have argued that a focus on the visceral materiality of food illuminates the “slipperiness” of care relations. Extending earlier work on materialities and agencies of eating (see Mol 2008; Probyn 2000), these analyses have particular resonance for a theorization of anorexic embodiment, extending the arguments about the mutability of material objects that have woven through the vignettes so far; here food as an object has, and makes, meaning through its presence as well as its absence across spaces.

During Lavis’ fieldwork in the EDU she noticed how participants often had difficulty even touching food; some put on rubber gloves to handle ingredients and others might nudge bits of broccoli or cheese around the chopping board with a spoon if they were charged with cooking their own dinner as part of treatment. Some would hold serving utensils by the tip, as far away as possible from the food and many would jump back in fear if a drop of mashed potato dripped off the spoon. The vignettes so far have highlighted how anorexic embodiment is relationally forged in encounters with other people—with their bodies, their gaze and their eating practices, for example. Participants’ fear of food extends that discussion by showing how making and unmaking *oneself* as anorexic, is a dynamic process enacted through the relationship between food and anorexia (Lavis 2013); this is seen through an attention to Hadia’s biscuits.

On the EDU every morning all the patients had to spend fifteen minutes in the dining room for morning snacks. The second of the highly regulated six meals and 4000 calories a day, morning snacks comprised milk with chocolate, strawberry or banana *Nesquik* and two biscuits. Those patients allowed weekend leave were expected to rigorously replicate this food timetable, including all snacks, whilst outside the EDU. Most participants admitted in interviews that even if they managed meals, snacks were “just too much to bear” as one participant, Cally, put it. Hadia, however, recounted how she always ate morning snacks on leave, if (as was usual) nothing else at all. Whilst at home for the weekend she would always walk to the same *Starbucks,* a mile or so from her home, and order the same latte and small pre-sealed packet of biscuits. She would then sit with these for two hours, sipping and nibbling, slowly feeling her anorexia, as she put it, “rise up around [her]”. The mechanics of this rising are intriguing; Hadia described how the biscuits—as intermingled signifiers of both the agency of choosing and paying for food herself (and therefore being a “bad anorexic”, as she put it) and the messy materiality of butter and sugar—were utterly terrifying. By sitting in *Starbucks* every week Hadia drew *too close* to food to be comfortable, thereby echoing discussion above of participants’ experiences of the threat posed by food in the EDU. Yet, this discomfort played an important role in Hadia’s anorexic embodiment, or rather perhaps, her active embodiment of anorexia. She intentionally incited her terror of food precisely because, in leading her to *fear for* her anorexia, it ‘triggered’ an upsurge of the illness; it made, she felt, her anorexia stronger. In her adherence to the same latte and biscuits each week, Hadia mobilised anorexia in order to hold on to it, creating a “safe space” carved through the illness. As such, her biscuits might appear at first glance to transfer her treatment regimen to the home spaces of weekend leave, adhering to the rules of the EDU and honouring her clinicians’ trust, as Eli put it. Yet, through them Hadia resisted their clinical conceptualisations of anorexia and selfhood. She felt these moments in *Starbucks* to release her anorexia from the grip of the EDU, thereby symbolically also shifting herself from that unwanted enclosed space to the other space offered, perhaps ambivalently, by anorexia; biscuits afforded Hadia a modality of “stepping out” of clinical conceptualisations and spaces. This echoes Kate’s reading of memoirs, which created what she felt to be an “anorexic space” within the space of the EDU, carving her own sense of everyday embodiment into the fabric of the patients’ lounge. Extending that discussion -  that food emerges here as an object through which anorexia may be maintained both goes against habitual understandings of the illness and yet is also clearly shaped by Hadia’s experiences of treatment. The biscuits were imbued with multiple meanings that did not necessarily connect to or even disrupt one another. Rather, for this moment in *Starbucks* they came powerfully to afford Hadia’s anorexic embodiment, recreating and renewing the “safe space” that she felt her illness to signify.

Across the vignettes, anorexic embodiment has been seen to be forged through the clinic’s ordering of space, time and materiality—an ordering which becomes absorbed into, but also reconfigured by, individuals’ experiences. As such, what Hadia’s biscuits draw to the fore is that all the material engagements in this paper have been about far more than the linearity of resisting treatment. In their mutability and movement across spaces and boundaries, the objects have shown how anorexic embodiment is forged through the fraught and mobile relationships between the clinical and the mundane. In both treatment and domestic spaces, these are carved into one another in ways that problematize medicalised understandings of the relationship between the body and anorexia. In so doing, these material engagements have challenged an arguably pervasive privileging of discourse over materiality, showing how bodies and the objects and spaces they encounter develop through mutually productive processes.

## Conclusions and Implications for Treatment

To date little scholarly attention has been paid to how materialities shape intimate experiences of mental ill-health. In interrogating how material objects and spaces forge, frame and disrupt what we term ‘anorexic embodiment’, this paper has opened up a consideration of how material environments relate to eating disorders and mental health experiences more broadly. We have sought to ask what theoretical and therapeutic possibilities, trajectories, and limitations are engendered by exploring mental health, illness, and treatment through a critical lens of materiality.

Specifically, the objects explored in this paper have elucidated the central, but previously-overlooked, role of materiality in forging lived experiences of anorexia. Ensuing directly from participants’ narratives, objects and spaces emerged from both datasets as central to embodied practices of “being anorexic” (see Gooldin 2008) and the making of anorexic embodiment through material “webs of relations” (Law 2009, p. 141).

Across the vignettes, clinical objects were seen to transition into mundane spaces, inhabiting the everyday. In Grace’s case, memories of the ambivalence of inpatient life, and a remembered—and actively negotiated—anorexic embodiment, materialized through a photo album, leafed through in the sunny environment of her kitchen. Likewise, Hadia used the seemingly mundane but profoundly clinical object of biscuits to disrupt treatment and reaffirm her anorexic body and being. Drawing together the sensory affordances of objects and the (direct or indirect) possibilities for material interaction prescribed by the clinic, participants thereby creatively mobilized material engagements to assert modes of being in the world. As such, reflecting on anorexia through a lens of materiality has shown that objects and the spaces they move through are a crucial part of the production of anorexic embodiment as this comes into being, is threatened, and is remade within treatment.

Crucially, these analyses have also shown that this movement of objects is bidirectional. Everyday household objects—such as televisions, books, and chairs—featured centrally in ward spaces and practices, turning intimacy inside out (see also Warin 2010). These objects were consistently dislocated: upholstered chairs were used for confinement; a television set was placed in a surveillance room for the patients to watch programmes while they themselves were being watched. For Sara, a generic institutional chair became her collaborator in a post-meal surveillance *pas de deux*, an enactment of compliance with ward rules (staying seated) and simultaneous resistance (stretching her limbs, with the chair as platform), communicating and embodying ambivalence. Likewise, the exchange of memoirs and calorie counting books between patients was not only disruptive but also productive; within a clinical space, this exchange enacted alternative regimes of care defined in specifically non-clinical terms.

Thus, in the clinic, these objects conveyed the flow between personal and clinical space, their very familiarity contributing to an embodied sense of displacement and liminality. The vignettes demonstrated how, when moved into clinical spaces, the material everyday-ness of objects becomes a central actor in the making or resisting of ‘disordered’ embodiment. Within an eating disorders ward, where, as some participants explained, every movement and utterance may be conscripted into a clinical portrayal of the individual as anorexic or bulimic (Eli 2014a), these objects thereby came to signify and materialize identity and sociality.

In each direction of flow, therefore, objects were seen to migrate across spatial and ontological boundaries, mutably taking on new meanings as they traversed the categories of the clinical and mundane. Whilst each object was situated within treatment, the insights they afford go beyond this to illuminate how anorexia is lived and felt. Exploring a dual-directional flow of objects between the clinical and the mundane has offered a way of gaining novel insights into *both* the ‘body in treatment’ *and *anorexic embodiment more broadly. By illustrating how the clinic both constrains and makes possible experiences and expressions of anorexic embodiment, a materialist lens has revealed the conflicts, inconsistencies, contradictions, and suffering that lie at the heart of lived experience. In so doing, participants’ material engagements also speak to the wider literature on treatment resistance in eating disorders (Schmidt and Treasure 2006) and have therapeutic implications.

An engagement with materialities illuminates core aspects of anorexia obscured from medical and popular discourse. From a clinical perspective (APA 2013) anorexia is seen to arise through pathologised emotions, cognitions, and behaviours, and is actualised in underweight: a definition that reinforces the mind/body split. In contrast, by attending to anorexic embodiment, our analyses have underscored the therapeutic need to understand how anorexia is lived processually and precariously day-by-day, across the blurred boundaries of everyday and medicalised practices. Moreover, we recognise anorexia as part of the wider continuum of eating disorders, rather than as a discrete disorder (Fairburn, Cooper and Shafran 2003; Eli 2018). As such, we suggest that our insights extend across the spectrum of DSM-diagnosed eating disorders. We argue, then, that rather than reproduce the imagined modus operandi of eating disorders, wherein an individual’s ‘undue’ fears and misjudged body image create bodily disorder, it is crucial to recognise how interactions between persons and material objects illuminate the deep relationality of eating disorders, and the interweaving of eating disorders, identity, and experiences of the world. Against the background of the high rates of relapse in eating disorders, this understanding is crucial to designing appropriate interventions that take account of embodied complexity.

A materialist perspective critiques the frequent privileging of the visuality of the body over an individual’s voice in treatment regimes, to instead bring situated lifeworlds to the fore. In demonstrating the necessity of moving beyond a pervasive focus on the body to consider eating disorders as fundamentally related to the environments in which they are lived, such a perspective calls for a reconsideration of ‘the body in treatment’ to understand a person’s body as part of a network. Such an understanding would consider how human and non-human others, including objects and spaces, shape eating disorders and their embodiment.

In turn, this materialist perspective has implications for how relapse is conceptualised and understood. It provokes questions regarding what constitutes a ‘trigger’ or risk factor for relapse. Whilst clinically a focus is habitually placed on the triggering or even ‘contagious’ possibilities of other people—through seeing their bodies or hearing their comments, for example—a materialist perspective extends that consideration by asking how other elements of the environment might maintain or ‘trigger’ eating disorders. This necessitates consideration of how therapists might work with patients to alter their environment (where possible) or craft a personal response to macro-environmental changes, such as a global pandemic, which fundamentally alter both human and non-human relationality.

This analysis has thereby shown how, using the lens of objects and spaces, it becomes possible to elucidate the anorexic body as both phenomenologically lived and socio-medically constituted. In so doing, our paper has sought to extend clinical discussions of anorexia and demonstrate that moving towards a materialist account of eating disorders has profound implications for treatment.

## Footnotes

1 Whilst we recognise the term ‘patient’ as contested and acknowledge its use as potentially stigmatising, in this paper we use it to stay close to the language of our ethnographic field sites. In each of these, participants referred to themselves as patients whilst in treatment.

2 This quote also appears in Eli (2014a).

## References

[CR1] Abbots E.-J., Lavis A., Abbots E.-J., Lavis A. (2013). Contours of Eating: Mapping the Terrain of Body/Food Encounters. Why We Eat.

[CR2] Arcelus J., Mitchell A.J., Wales J., Nielsen S. (2011). Mortality Rates in Patients with Anorexia Nervosa and Other Eating Disorders. A Meta-analysis of 36 Studies. Archives of General Psychiatry.

[CR3] American Psychiatric Association (2013). Diagnostic and Statistical Manual of Mental Disorders (DSM-5®).

[CR4] Bardone-Cone A., Thompson K., Miller A. (2020). The Self and Eating Disorders. Journal of Personality.

[CR62] Bordo, S. (1993) *Unbearable Weight: Feminism, Western Culture, and the Body.* University of California Press.

[CR5] Braun V., Clarke V. (2013). Successful Qualitative Research: A Practical Guide for Beginners.

[CR6] Bray A. (1996). The Anorexic Body: Reading Disorders. Cultural Studies.

[CR8] Brownlie J., Spandler H. (2018). Materialities of Mundane Care and the Art of Holding One’s Own. Sociology of Health & Illness.

[CR9] Bruch H. (1973). Eating Disorders: Obesity, Anorexia Nervosa, and the Person Within.

[CR63] Burke E. (2006). Feminine Visions: Anoexia and Contagion in Pop Discourse. Feminist Media Studies.

[CR10] Bury M. (1982). Chronic Illness as Biographical Disruption. Sociology Health & Illness.

[CR11] Buse C., Martin D., Nettleton S. (2018). Conceptualising ‘Materialities of Care’: Making Visible Mundane Material Culture in Health and Social Care Contexts. Sociology of Health & Illness.

[CR61] Carter JC, Mercer-Lynn KB, Norwood SJ, Bewell-Weiss CV, Crosby RD, Woodside DB, Olmsted MP. (2012) A prospective study of predictors of relapse in anorexia nervosa: implications for relapse prevention. *Psychiatry Res, 200*(2–3), 518–523. 10.1016/j.psychres.2012.04.037 22657951

[CR12] Coole, D., and Frost, S. 2010 Introducing the New Materialisms. *In* New Materialisms: Ontology, Agency, and Politics. D. Coole and S. Frost, eds., pp. 1–43.

[CR13] Csordas T. (1993). Somatic Modes of Attention. Cultural Anthropology.

[CR14] Demmler J.C., Brophy S.T., Marchant A., John A., Tan J.O. (2020). Shining the Light on Eating Disorders, Incidence, Prognosis and Profiling of Patients in Primary and Secondary Care: National Data Linkage Study. British Journal of Psychiatry.

[CR15] Desjarlais R., Throop C.J. (2011). Phenomenological Approaches in Anthropology. Annual Review of Anthropology.

[CR16] DeSilvey C. (2006). Observed Decay: Telling Stories with Mutable Things. Journal of Material Culture.

[CR18] Eli, K. 2014a. Between Difference and Belonging: Configuring Self and Others in Inpatient Treatment for Eating Disorders. PLoS ONE 9(9): e105452. 10.1371/journal.pone.0105452 PMC4161313 25210886

[CR19] Eli, K. 2014b. An Embodied Belonging: Amenorrhea and Anorexic Subjectivities. Medicine Anthropology Theory 1(1): 53–80.

[CR20] Eli K. (2016). “The Body Remembers”: Narrating Embodied Reconciliations of Eating Disorder and Recovery. Anthropology & Medicine.

[CR21] Eli K. (2018). Striving for Liminality: Eating Disorder and Social Suffering. Transcultural Psychiatry.

[CR22] Ellis J. (2018). Family Food Practices: Relationships, Materiality and the Everyday at the End of Life. Sociology of Health and Illness.

[CR23] Fairburn C.G., Cooper Z., Shafran R. (2003). Cognitive Behaviour Therapy for Eating Disorders: A “Transdiagnostic” Theory and Treatment. Behaviour Research and Therapy.

[CR24] Gibson D., Workman C., Mehler P.S. (2019). Medical Complications of Anorexia Nervosa and Bulimia Nervosa. Psychiatric Clinics of North America.

[CR26] Gooldin S. (2008). Being Anorexic. Medical Anthropology Quarterly.

[CR27] Gremillion H. (2003). Feeding Anorexia: Gender and Power at a Treatment Center.

[CR28] Henare A., Holbraad M., Wastell S. (2007). Thinking Through Things: Theorising Artefacts Ethnographically.

[CR29] Heywood L. (1996). Dedication to Hunger: The Anorexic Aesthetic in Modern Culture.

[CR30] Khalsa S.S., Portnoff L.C., McCurdy-McKinnon D., Feusner J.D. (2017). What Happens After Treatment? A Systematic Review of Relapse, Remission, and Recovery in Anorexia Nervosa. Journal of Eating Disorders.

[CR31] LaMarre A., Rice C. (2021). Recovering Uncertainty: Exploring Eating Disorder Recovery in Context. Culture, Medicine, Psychiatry.

[CR32] Lavis, A. 2011 The Boundaries of a Good Anorexic: Exploring Pro-anorexia on the Internet and in the Clinic. Doctoral thesis. Goldsmiths, University of London. http://eprints.gold.ac.uk/6507/.

[CR33] Lavis A., Abbots E.-J., Lavis A. (2013). The Substance of Absence: Exploring Eating and Anorexia. Why We Eat.

[CR34] Lavis A., Abbots E.-J., Lavis A., Attala L. (2015). Careful Starving: Exploring (not) Eating, Care and Anorexia. Careful Eating: Bodies.

[CR35] Lavis A. (2016). Foods, Bodies and the Stuff of (Not) Eating in Anorexia. Gastronomica.

[CR36] Lavis A. (2018). Not Eating or Tasting Other Ways to Live: a Qualitative Analysis of ‘Living Through’ and Desiring to Maintain Anorexia. Transcultural Psychiatry.

[CR37] Lavis A., Abbots E.-J., Attala L., Abbots E-J, Lavis A., Attala L. (2015). Reflecting on the Embodied Intersections of Eating and Caring. Careful Eating: Bodies.

[CR38] Lavis, A., and Eli, K. (2016) Editorial—Corporeal: Exploring the Material Dynamics of Embodiment. M/C. Journal of Media and Culture 19(1).

[CR64] Law, J. (2009) Actor network theory and material semiotics. In Turner, B. S. (ed.) *The New Blackwell Companion to Social Theory*. Chichester, Malden, MA: Wiley-Blackwell.

[CR39] Lester R. (1997). The (Dis) Embodied Self in Anorexia Nervosa. Social Science & Medicine.

[CR40] Lester R. (2007). Critical Therapeutics: Cultural Politics and Clinical Reality in Two Eating Disorder Treatment Centers. Medical Anthropology Quarterly.

[CR41] Lester R. (2019). Famished: Eating Disorders and Failed Care in America.

[CR42] Low S.M. (2003). Embodied Space (S) Anthropological Theories of Body, Space, and Culture. Space and Culture.

[CR43] Malafouris L. (2019). Understanding the Effects of Materiality on Mental Health. British Journal of Psychology Bulletin.

[CR44] Malson H., Ussher J.M. (1996). Body Poly-texts: Discourses of the Anorexic Body. Journal of Community & Applied Social Psychology.

[CR45] Miller D. (2010). Stuff.

[CR46] Miller D., Woodward S. (2012). Blue Jeans: The Art of the Ordinary.

[CR47] Mol A. (2008). I Eat an Apple: On Theorizing Subjectivities. Subjectivity.

[CR48] Olsen B. (2003). Material Culture after Text: Re-Membering Things. Norwegian Archeological Review.

[CR49] Parish J. (2018). Obsessive Compulsive Disorder (OCD): The ritual Moment of Social Death. Anthropology Today.

[CR50] Pink S., Morgan J., Dainty A. (2014). The Safe Hand: Gels, Water, Gloves and the Materiality of Tactile Knowing. Journal of Material Culture.

[CR51] Probyn E. (2000). Carnal Appetites: Food Sex Identities.

[CR52] Saukko P. (2000). Between Voice and Discourse: Quilting Interviews on Anorexia. Qualitative Inquiry.

[CR53] Schmidt U., Treasure J. (2006). Anorexia Nervosa: Valued and Visible. A Cognitive-Interpersonal Maintenance Model and its Implications for Research and Practice. British Journal of Clinical Psychology.

[CR54] Schmidt U., Adan R., Böhm I., Campbell I.C., Dingemans A., Ehrlich S., Elzakkers I., Favaro A., Giel K., Harrison A., Himmerich H. (2016). Eating Disorders: The Big Issue. The Lancet Psychiatry.

[CR55] Silver, J.M. (director) 2003 Hunger Point. United States: Jaffe/Braunstein Films.

[CR65] Tan, J., Stewart, A., Fitzpatrick, R. & Hope, A. (2006) Competence to Make Treatment Decisions in Anorexia Nervosa: Thinking Processes and Values. *Philosophy, Psychiatry, & Psychology,* 13, 267–282. 10.1353/ppp.2007.0032 PMC2121578 18066393

[CR56] Treasure J., Nazar B.P. (2016). Interventions for the Carers of Patients with Eating Disorders. Current Psychiatry Reports.

[CR57] Warin M. (2003). Miasmatic Calories and Saturating Fats: Fear of Contamination in Anorexia. Culture, Medicine and Psychiatry.

[CR59] Warin M. (2010). Abject Relations: Everyday Worlds of Anorexia.

[CR60] Whitman, W. (1975 [1855]) Song of myself. *In* The Complete Poems. F. Murphy, ed., pp. 63–124. London: Penguin Books.

